# Effects of 1-methylcyclopropene and high-voltage electrostatic field treatment on postharvest storage quality of Korla fragrant pears

**DOI:** 10.1016/j.fochx.2025.103457

**Published:** 2025-12-27

**Authors:** Yujiao Zhang, Chengxin Fei, Ruojie Zhao, Oujun Dai, Zhixiong Deng, Bei Fan, Duoyong Zhao, Fengzhong Wang, Yatao Huang

**Affiliations:** a Xinjiang Key Laboratory of Agro-products Quality & Safety, Laboratory of Quality & Safety Risk Assessment for Agro-Products(Urumqi), Ministry of Agriculture and Rural Affairs, Urumqi 830091, China; b Institute of Western Agriculture, the Chinese Academy of Agricultural Sciences/Xinjiang Engineering Technology Research Center for Exploration and Quality Evaluation of Characteristic Agricultural and Livestock Product Resources, Changji 831100, China; c Institute of Food Science and Technology, Chinese Academy of Agricultural Sciences/Laboratory of Risk Assessment for Processed Agro-food Quality and Safety, Ministry of Agriculture (Beijing), Beijing 100193, China

**Keywords:** Korla fragrant pear, 1-methylcyclopropene, High-voltage electrostatic field, Postharvest quality, Metabolomics

## Abstract

This study investigated the effects of combined 1-methylcyclopropene (1-MCP) and high-voltage electrostatic field (HVEF) treatment on the postharvest quality of Korla fragrant pears using physicochemical and metabolomic analyses. The combined treatment significantly suppressed respiration and ethylene production, better maintained firmness, and reduced weight loss compared to controls. Volatile metabolomics identified 1301 compounds, with esters and terpenoids being most abundant. Enrichment analysis highlighted phenylpropanoid and sesquiterpenoid biosynthesis as key pathways. Sensory evaluation confirmed the treatment enhanced balsamic and citrus notes while preserving the typical flavor profile. These findings demonstrate that 1-MCP + HVEF treatment effectively maintains pear quality during long-term storage by modulating physiological and aroma-related metabolic pathways.

## Highlights

• 1-MCP + HVEF treatment significantly reduced ethylene release rate, weight loss in Korla fragrant pears.
• Terpenoids and esters are key metabolites determining flavor differences in late storage.
• Sesquiterpenoid and triterpenoid biosynthesis, phenylpropanoid biosynthesis are the most critical metabolic pathways.
• After 80d storage, 1-MCP + HVEF enhanced balsamic and citrus flavors while reducing fatty and fresh notes.
• 1-MCP + HVEF increased beneficial flavor compounds, such as esters and terpenoids, during long-term storage.

## Graphical abstract

**Figure:**
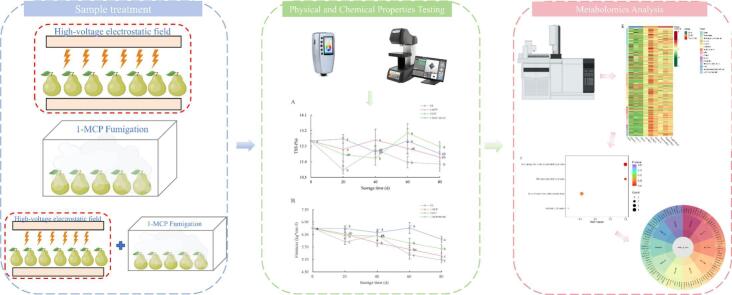
Graphic Abstract

## 1 Introduction

The Korla fragrant pear, an ancient indigenous variety originating from Xinjiang, is highly appreciated by consumers for its distinctive flavor, texture, and rich nutritional profile, which includes soluble sugars, amino acids, phenols, flavonoids, organic acids, and dietary fiber (Fang et al., 2023). The methods used to analyze fruit components, such as the extraction and characterization of dietary fiber from other fruits like Rubus chingii, are crucial for a comprehensive understanding of fruit quality and functional properties (Wang et al., 2022). After harvest, fruits continue to undergo active metabolism. During postharvest physiological activities, enzymatic oxidation pathways such as respiration drive continuous material transformation in fruit tissues. The synergy between endogenous enzyme systems and microbial communities during this process triggers a series of physiological changes, including altered cell membrane permeability, leaching of soluble solids, and disassembly of cell wall structures (Wei et al., 2025). These changes ultimately lead to quality deterioration in postharvest fruits, manifested as nutrient loss, flavor dissipation, texture degradation, and increased spoilage indices. Fruits can be categorized into climacteric and non-climacteric types based on respiratory behavior. Climacteric fruits exhibit a distinct ripening phase accompanied by an ethylene burst, marking the transition from maturation to senescence and resulting in phased quality decline. In contrast, non-climacteric fruits enter senescence directly after harvest, showing continuous quality deterioration (Fukano & Tachiki, 2021). In summary, postharvest quality loss in fruits—driven by microorganisms, enzymes, ethylene, and other factors—significantly reduces their edible value and commercial shelf life, leading to substantial economic losses. Therefore, maintaining postharvest fruit quality and extending shelf life remain urgent challenges in the fruit and vegetable industry.

Ethylene, an endogenous plant hormone, acts as a key regulator of fruit ripening and senescence. Currently, 1-methylcyclopropene (1-MCP) treatment has become an effective method for inhibiting ethylene action in climacteric fruits. As an ethylene action inhibitor, 1-MCP competitively and irreversibly binds to ethylene receptor proteins, blocking ethylene signal transduction and effectively delaying postharvest ripening and senescence, thereby extending shelf life (Srividhya et al., 2023). 1-MCP is often combined with other postharvest preservation technologies—such as KMnO₄ and modified atmosphere packaging (Maqbool et al., 2024; Patiño et al., 2018)— to better suppress ethylene production rates. Furthermore, by modulating the activity of cell wall-degrading enzymes, this treatment effectively retards chlorophyll degradation, reduces respiratory intensity, and suppresses metabolic activities associated with internal browning, thereby preserving fruit quality and extending the storage life. Additionally, existing studies indicate that 1-MCP can modulate the biosynthesis of volatile aroma compounds, thereby influencing fruit flavor quality (Cai et al., 2019).

In recent years, HVEF has gained increasing attention in fruit preservation due to its non-thermal effects, high efficiency, low energy consumption, residue-free operation, and cost-effectiveness (Nie et al., 2024). It has been reported that HVEF influences the intrinsic electric field of fruits (Dalvi-Isfahan et al., 2016). Gas discharge ionisation during HVEF treatment generates reactive oxygen species (e.g., ozone) and charged particles, which can regulate stomatal aperture, suppress sugar metabolism pathways, and thereby help maintain postharvest quality (Lotfi et al., 2022). These oxidative active substances can disrupt microbial membranes integrity, thereby inhibiting microbial growth. Similar germicidal effects of induced electric fields on resilient bacterial spores, such as *Geobacillus stearothermophilus*, *Escherichia coli* have been reported in liquid food models, further supporting the potential of electric field-based technologies in food preservation (Xue et al., 2025). Moreover, the applied electric field may influence cell membrane potential, slowing cellular metabolism and water loss (Valdez-Miranda et al., 2024). Additionally, these reactive species inhibit the activity of ripening-related enzymes (e.g., polyphenol oxidase and cellulase) (Liu et al., 2017), thereby delaying fruit softening and extending shelf life.

Evidence indicates that both 1-Methylcyclopropene (1-MCP) and High-Voltage Electrostatic Field (HVEF) treatments individually contribute to the regulation of fruit metabolic processes and maintenance of postharvest quality. However, existing studies have primarily focused on their independent applications or combinations with other preservation technologies, while the synergistic potential of combining 1-MCP with HVEF remains largely unexplored, particularly in Korla fragrant pears. Specifically, it remains unclear how their combined application influences the physicochemical properties and volatile metabolite profiles during long-term cold storage. This study aims to address this knowledge gap by systematically evaluating the effects of combined 1-MCP and HVEF treatment on the quality and volatile metabolome of Korla fragrant pears. We hypothesize that the dual treatment may operate through complementary mechanisms—1-MCP blocking ethylene signal transduction and HVEF modulating respiratory metabolism and microbial activity—thereby more effectively delaying senescence and preserving aromatic compounds. This work pioneers a novel preservation strategy that not only offers a practical approach for quality maintenance but also reveals new insights into the underlying metabolic regulation. The findings are expected to facilitate the application of this combined technology for improving the storage quality of Korla fragrant pears and provide a theoretical foundation for understanding its mechanistic basis.

## 2 Materials and methods

### 2.1 Plant material, treatment, and storage condition

This study employed commercially mature “Korla fragrant pears” harvested from experimental orchards in Bayingolin Mongol Autonomous Prefecture. Immediately post-harvest, the fruit was transported under cold chain conditions to the cold storage facility of the Processing Institute, Xinjiang Academy of Agricultural Sciences. Following a 6-h pre-cooling period at 4 °C, fruit exhibiting uniform size, consistent colouration, and free from physiological disorders, mechanical damage, or pest infestation was selected for use. The fruits were randomly divided into four groups, each with three replicates comprising 80 fruits per replicate. Treatment protocols were as follows: (1) CK group: control (no treatment applied); (2) 1-MCP group: fumigated with 1.0 μL/L 1-methylcyclopropene in a sealed polystyrene box for 12 h; (3) HVEF group: continuous treatment within a plastic tent equipped with a high-voltage electrostatic field system (2500 V, 50 Hz); (4) 1-MCP + HVEF group: fumigation as per the 1-MCP group followed by electrostatic field treatment as per the HVEF group. The high-voltage electrostatic field plates maintained a non-contact distance of 15 cm from the fruit. All fruits were stored for 80 days at 0 ± 0.5 °C with relative humidity of 85–90 %. Random samples were collected from each group every 20 days for analysis. All experiments were replicated three times.

### 2.2 Assessment of fruit quality attributes

#### 2.2.1 Determination of fruit weight loss rate

To determine the fruit weight loss rate, six fruits were randomly sampled from each group. Fruit weight was recorded at 20d intervals. The weight loss rate was calculated using the following formula, with results expressed as a percentage:

$$\text{Weight loss}\left(\%\right)=\frac{\text{Original weight}-\text{subsequent wieght}}{\text{Original weight}}\times 100$$

#### 2.2.2 Fruit firmness measurement

Measurements were taken using a GY-4 digital fruit firmness tester (probe diameter: 2 mm), six fruits were randomly sampled from each group. For each group, three equidistant points were selected on the equatorial region of Korla fragrant pears, peeled, and measured. Each measurement was repeated three times.

#### 2.2.3 Determination of respiratory rate and ethylene release rate

For this purpose, an F-950 portable gas analyzer was employed, six fruits were randomly sampled from each group. Respiratory rate and ethylene release rate were measured using the static method: pear fruits were placed in a 4.4 L respiration chamber and left at room temperature for 2 h before analysis by the gas analyzer. Each determination was repeated three times per group.

#### 2.2.4 Determination of Total soluble solids (TSS) and titratable acidity (TA)

TSS and TA content were measured using a PAL-BX|ACID F5 sugar-acidity analyzer. Six fruits were randomly sampled from each group, cored, homogenized, filtered, and 3 mL of filtrate was analyzed. Each group underwent three replicates.

#### 2.2.5 Color measurement

Fruit samples were washed, dried, and analyzed using an LS175 color difference meter. Six fruits were randomly selected from each group for measurement, with weighing conducted every 20 days. Three distinct positions on the equatorial region of each fruit were examined, recording the L*, a*, b* values, and ΔE. Measurements were repeated three times. In this context, L* represents lightness, a* indicates redness, b* denotes yellowness, and ΔE signifies the total color difference.

### 2.3 Ultra-fast gas chromatography electronic nose analysis

For the electronic nose analysis, six fragrant pear fruits were randomly selected from each group, chopped, and 1–2 g of sample was weighed into a 20 mL headspace vial, which was then sealed. Each group was analyzed in five replicates. The incubation was carried out at 50 °C for 30 min with agitation at 500 r/min. A volume of 3000 μL of headspace gas was injected at a rate of 125 μL/s into an injector maintained at 200 °C, and the injection duration was 29 s. The trap was initially held at 50 °C with a split flow of 10 mL/min, and the trapping time was 34 s; thereafter, the trap temperature was increased to 240 °C. The capillary column was initially set at 50 °C and then programmed to rise at 2 °C/s to 250 °C. The total acquisition time was 110 s. The detector temperature was set at 260 °C, and the FID signal gain was set to 12.

### 2.4 Qualitative and quantitative analysis of volatile metabolites in Korla fragrant pears

#### 2.4.1 Sample extraction for GC–MS/MS analysis

Volatile metabolites were extracted and qualitatively/quantitatively analyzed by Wuhan Maiwei Metabolic Biotechnology Co., Ltd. according to their standardised protocol. Briefly, pear fruit samples stored at −80 °C were ground in liquid nitrogen using a ball mill (MM400, Retsch) for 1.5 min. Five hundred milligrams of the powdered sample were weighed into 20 mL headspace vials. Each vial received 2 mL saturated NaCl solution, 20 μL (10 μg/mL) 3-hexanone-2,2,4,4-d4 (CAS No.: 24588–54-3) solution as internal standard, and was sealed with a silica cap. For Solid-Phase Microextraction (SPME): Employ a 120 μm DVB/CWR/PDMS extraction head for headspace solid-phase microextraction (HP-SPME) at 60 °C for 15 min. Following extraction, volatiles were desorbed at the GC instrument (Model 8890; Agilent Technologies, Palo Alto, California, USA) at 250 °C for 5 min. VOCs were identified using an Agilent 8890 GC coupled with a 7000D mass spectrometer (Palo Alto, California, USA; Agilent Technologies). Helium was employed as the carrier gas at a constant flow rate of 1.2 mL/min. The injection port temperature was maintained at 250 °C with a solvent delay of 3.5 min. The temperature programme was set as follows: 40 °C held for 3.5 min, followed by a 10 °C/min ramp to 100 °C, then a 7 °C/min ramp to 180 °C, and finally a 25 °C/min ramp to 280 °C, held for 5 min. All mass spectra were acquired in electron-impact ionisation (EI) mode at an ionisation voltage of 70 eV. Ion source, quadrupole, and mass spectrometer interface temperatures were 230 °C, 150 °C, and 280 °C, respectively. The scanning mode employed was selected ion monitoring (SIM), with qualitative and quantitative ion precision scanning (GB 23200.8–2016). Mass spectrometry was compared with MWGC and linear retention indices to identify and separate volatile compounds. Concurrently, quality control (QC) samples prepared from 10 g mixtures of each sample were analyzed.

#### 2.4.2 Qualitative and quantitative analysis of metabolites

Raw data from mass spectrometry analysis were processed using MassHunter software for qualitative and quantitative analysis. Following the methodology outlined in the reference, the relative content of VOCs in the samples was calculated using the following formula:

$$X_{i}=\frac{V_{s}\times C_{s}}{M}\times \frac{I_{i}}{I_{s}}\times 10^{-3}$$

X_i_ denotes the content of compound i in the test sample (μg/g); V_s_ represents the volume of internal standard added (μL); C_s_ indicates the concentration of the internal standard (μg/mL); M signifies the mass of the test sample (g); I_s_ denotes the peak area of the internal standard; and I_i_ indicates the peak area of compound i in the test sample.

#### 2.4.3 Relative odour activity value (ROAV) analysis

The relative odour activity value (ROAV) primarily serves to elucidate the contribution of each aroma compound to the overall flavor profile of a sample. It is chiefly employed to identify key flavor compounds in various foodstuffs. Its formula is as follows:

$${\text{ROAV}}_{i}=\frac{c_{i}}{T_{i}}$$

where C and T denote the relative content and threshold of compound i, respectively.

### 2.5 Statistical analysis

Data processing was conducted using SPSS 22.0 software, with Duncan's multiple range test employed to assess significant differences (*p* < 0.05). Correlation analysis and visualization were performed using Excel 2010. Analyses including PCA, HCA, Venn diagrams, volcano plots, flavor wheels, and K-means clustering were completed via the Maiwei Cloud platform (https://cloud.metware.cn). Metabolites were annotated via the KEGG Compound database and mapped to KEGG metabolic pathways.

## 3 Results and discussion

### 3.1 Weight loss

**Fig. 1:**
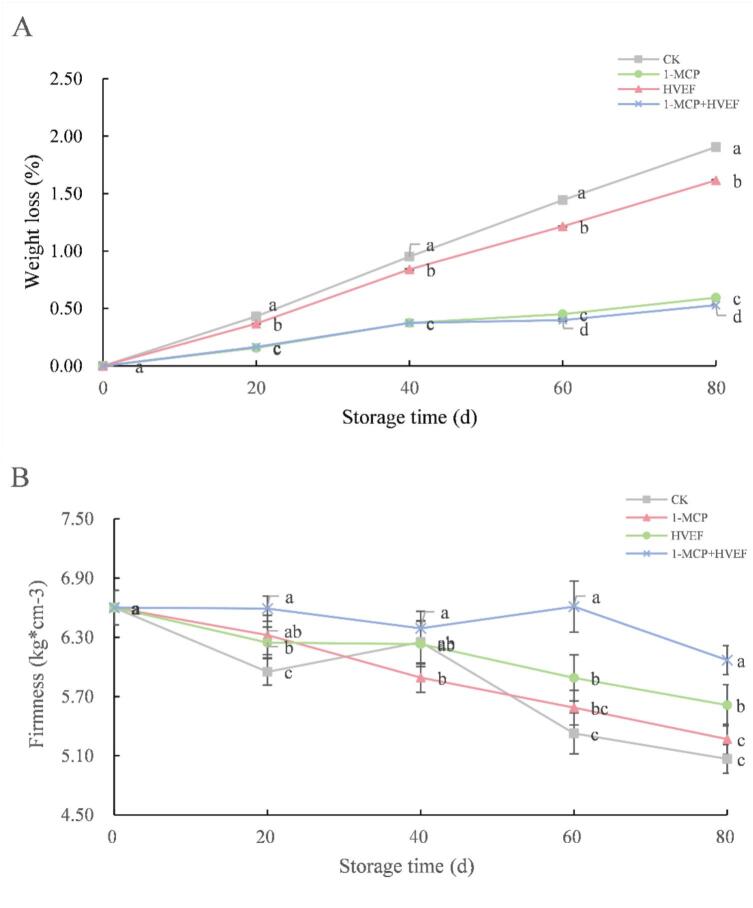
Effects of 1-MCP, HVEF, and 1-MCP + HVEF on fruit weight loss (A), firmness (B).

Weight loss during postharvest storage primarily results from transpiration and respiration, with transpiration accounting for over 97 % of total weight loss in fruits (Díaz-Pérez et al., 2007). Excessive weight loss leads to fruit shriveling and wilting, adversely affecting flavor quality. As shown in Fig. 1A, weight loss gradually increased across all groups during storage. Compared to the control, both the 1-MCP and combined treatment groups effectively suppressed weight loss throughout the storage period. At each sampling point from 0 to 80 days, the combined treatment consistently resulted in the lowest weight loss, indicating a synergistic effect beyond that achieved by either treatment alone. This may be attributed to the ability of 1-MCP to inhibit ethylene production, thereby delaying normal fruit maturation and reducing metabolic activity in pears. Consistent with previous studies (Li et al., 2022; Liu et al., 2017). A proposed mechanism is that HVEF alters the transmembrane potential, facilitating the movement of charged particles (e.g., calcium ions) and generating bioelectrical signals that regulate metabolic enzyme activity and maturation-related processes (Dalvi-Isfahan et al., 2016). Furthermore, both 1-MCP and HVEF significantly reduced respiration intensity in Korla fragrant pears (Fig. 1A), which further contributed to limiting weight loss. The synergistic suppression of respiration likely represents a key mechanism through which the combined 1-MCP and HVEF treatment mitigates postharvest weight loss.

### 3.2 Firmness

Firmness serves as a key indicator of fruit freshness and overall quality. Higher firmness values generally reflect slower metabolic rates and reduced water evaporation, both characteristic of well-preserved postharvest fruit. During storage, although firmness showed a general declining trend across all groups, the treated samples maintained significantly higher firmness than the control. At each sampling point from 0 to 80 days, the combined 1-MCP + HVEF treatment resulted in the highest firmness retention, with no significant difference from initial values after 80 days of storage (Fig. 1B). Consistent with previous research findings (Liu et al., 2017). Fruit softening during ripening is closely associated with water loss and cell wall thinning. The combined application of 1-MCP and HVEF suppressed endogenous ethylene biosynthesis, consequently delaying the respiratory climacteric. This inhibitory effect was directly reflected in reduced β-amylase activity, leading to minimal starch degradation and the highest fruit firmness among all groups. Furthermore, the limited breakdown of complex compounds better preserved cell wall structure, contributing to maintained firmness (Rahman et al., 2023). These results demonstrate that the 1-MCP + HVEF combination effectively preserves the appearance and structural integrity of fruit samples. Importantly, our findings indicate that the combined treatment more effectively delays firmness loss compared to either 1-MCP or HVEF applied individually.

### 3.3 Respiratory rate

**Fig. 2:**
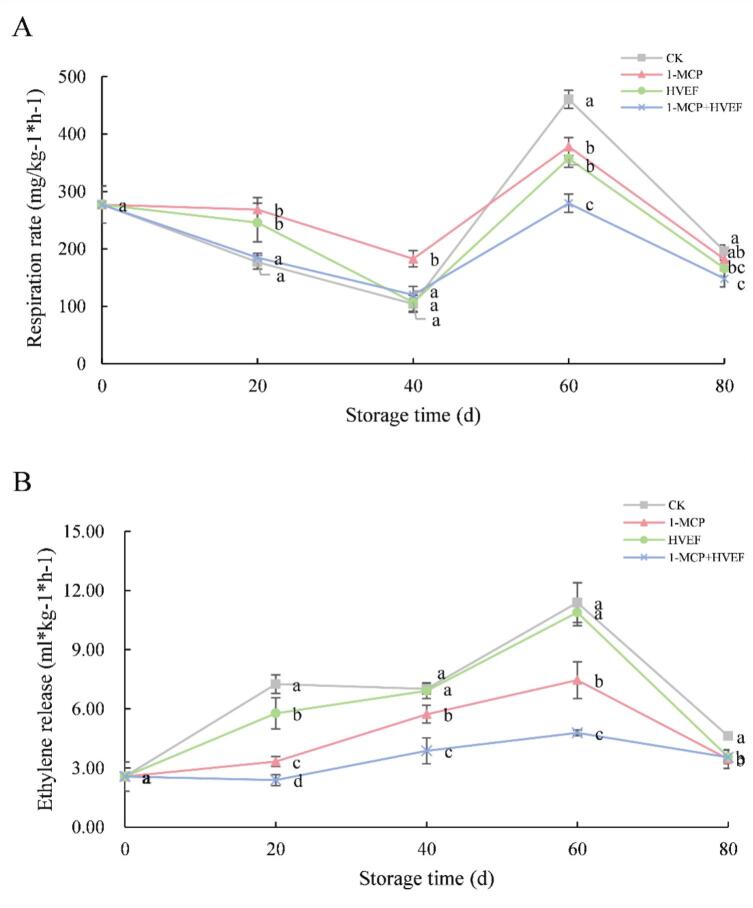
Effects of 1-MCP, HVEF, and 1-MCP + HVEF on fruit ethylene release (A) and respiration rate (B).

Ethylene biosynthesis and the respiratory climacteric are key physiological processes regulating fruit maturation and senescence. Increased respiration rates and ethylene production characterize the later stages of fruit ripening, primarily driven by continuous respiration associated with chlorophyll degradation and starch breakdown (Wang et al., 2015). Higher respiration rates accelerate nutrient consumption and shorten the postharvest lifespan of fruit (Navarro et al., 2006). Therefore, controlling respiration during storage is critical for extending the potential shelf life of fruits and vegetables. As shown in Fig. 2A, the respiration rate of Korla fragrant pears upon entry into storage after pre-cooling was 277.37 mg CO₂/kg·h. Throughout the 80-day storage period, respiration rates in all groups generally followed a pattern of initial decline, followed by an increase and subsequent decrease, peaking on day 60. The initial sharp decrease in respiration may be attributed to the rapid temperature reduction. Compared to the control, all three treatments partially suppressed the increase in respiration during storage. By day 40, respiration rates were lower than those measured on day 20, followed by a sharp increase to the peak on day 60, and a marked decline in the final 20 days. Notably, after 80 days of storage, the 1-MCP + HVEF combination group exhibited the lowest respiration rate among all groups, demonstrating a synergistic inhibitory effect on respiration. Reportedly, 1-MCP acts as a competitive inhibitor of ethylene receptors, binding to these receptors and suppressing the ethylene signaling pathway, thereby delaying respiratory metabolism (Srividhya et al., 2023). HVEF, on the other hand, induces conformational distortion in the Fe^3+^ coordination center of iron-containing oxidases, leading to catalytic inactivation and disruption of the electron transport chain in respiration (Zhang et al., 2022). Additionally, ozone-mediated regulation of stomatal movement can inhibit respiratory metabolism and reduce transpiration rates in fruits and vegetables. The present findings confirm that the combined 1-MCP and HVEF treatment effectively suppresses fruit respiration through distinct yet complementary mechanisms.

### 3.4 Ethylene release

As shown in Fig. 2B, ethylene production in Korla fragrant pears exhibited an initial increase followed by a gradual decline throughout the storage period. All three treatments significantly suppressed ethylene release over the 80-day storage duration compared to the control group. Among them, the 1-MCP-treated group consistently showed lower ethylene emission than both the control and HVEF-treated groups. Notably, the combined 1-MCP + HVEF treatment was more effective in reducing ethylene release than either treatment applied individually. These findings align with previous reports: Chang et al. (2023) observed that HVEF treatment effectively reduced ethylene synthesis and maintained quality in jujube fruit, while 1-MCP application has been shown to inhibit ethylene production in grapes (Shi et al., 2023). A plausible explanation is that HVEF may suppress enzymatic activity, alleviate cellular tissue damage, and lower respiratory intensity, thereby reducing endogenous ethylene levels in fruit (Liu et al., 2017). This mechanism could account for the diminished ethylene release observed in HVEF-treated pears in the present study. Moreover, the reduced endogenous ethylene following HVEF exposure may enhance the binding of 1-MCP to its receptor sites, leading to more effective suppression of ethylene biosynthesis—a synergistic effect supported by the results of the combined treatment.

### 3.5 Total soluble solids (TSS)

**Fig. 3:**
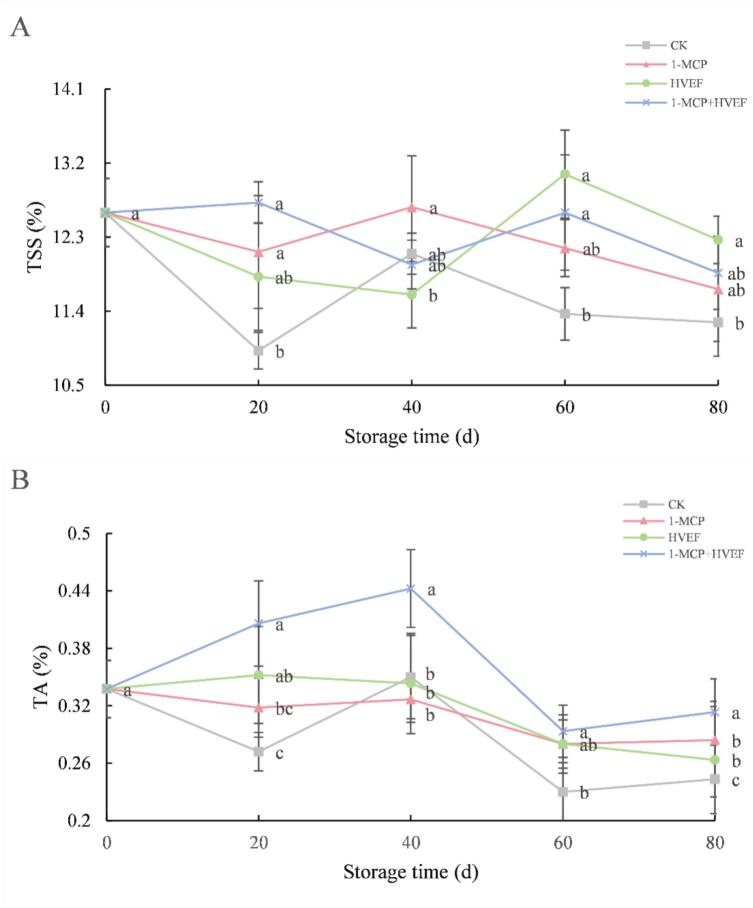
Effects of 1-MCP, HVEF, and 1-MCP + HVEF on fruit TSS (A), and TA (B).

Total soluble solids (TSS) and titratable acidity (TA) are key parameters influencing the taste and flavor of fruits. As the primary substrate for respiration, TSS comprises soluble compounds such as sugars, organic acids, and vitamins, with higher levels generally indicating advanced maturity (Lin et al., 2020). Minimal fluctuation in TSS during storage reflects better fruit preservation. As shown in Fig. 3A, TSS content in Korla fragrant pears pulp exhibited an initial decrease, followed by an increase and subsequent decline during storage. The early reduction may be attributed to low temperature stress, which temporarily suppressed respiratory metabolism while TSS was rapidly consumed as a direct energy source at a rate exceeding the hydrolysis of complex carbohydrates. The subsequent increase likely resulted from the degradation of macromolecules such as starch into soluble sugars during postharvest maturation. However, prolonged storage accelerated fruit senescence and respiration, leading to a pronounced decline in TSS (Ruelas-Chacon et al., 2017). Notably, the combined 1-MCP + HVEF treatment significantly attenuated TSS fluctuations, indicating slower production and consumption of soluble solids. This suggests that the combined treatment effectively suppressed respiration and delayed pear maturation. While the control and 1-MCP-treated groups reached peak TSS at day 40, the HVEF and combined treatment groups peaked at day 60. This delay may be explained by the inhibitory effect of HVEF on electron transport in the respiratory chain, which reduces respiratory rate and physiological metabolism. Throughout storage, all treatment groups maintained higher TSS levels than the control, indicating that 1-MCP + HVEF helps mitigate respiration, transpiration, and endogenous enzyme activity, thereby preserving cell wall structure and internal substance stability (Lou et al., 2024). These findings demonstrate that the combined 1-MCP + HVEF treatment effectively delays nutrient depletion in Korla fragrant pears during storage.

### 3.6 Tartaric acid (TA)

Titratable acidity (TA) serves as a substrate for respiration and typically declines during fruit maturation (Abdipour et al., 2020). In this study, TA content in 1-MCP- and HVEF-treated pears remained relatively stable during the first 40 days of storage, followed by a gradual decrease. In contrast, the combined 1-MCP + HVEF treatment led to an initial increase in TA, which subsequently declined (Fig. 3B). Although both individual treatments delayed TA reduction, all groups exhibited a significant decrease in TA by day 40 (p<0.05). The decline in TA is primarily attributed to the consumption of organic acids through physiological activities such as respiration (Jiang et al., 2018), which becomes more pronounced as storage progresses. These observations align with previous reports (Zhou et al., 2024). This phenomenon may be attributed to the suppression of respiratory intensity in Korla fragrant pears by 1-MCP and HVEF treatments, which subsequently slows the degradation of complex compounds and delays the decline in titratable acidity. Specifically, 1-MCP inhibits ethylene signaling and respiratory metabolism, while HVEF disrupts electron transport in the respiratory chain, collectively reducing the catabolic utilization of organic acids and contributing to better TA retention during storage. Throughout the storage period, the control group showed significantly lower TA content compared to the combined treatment group (p<0.05). These results demonstrate that the 1-MCP + HVEF combination more effectively maintains TA levels in Korla fragrant pears during extended storage.

### 3.7 Color

**Fig. 4:**
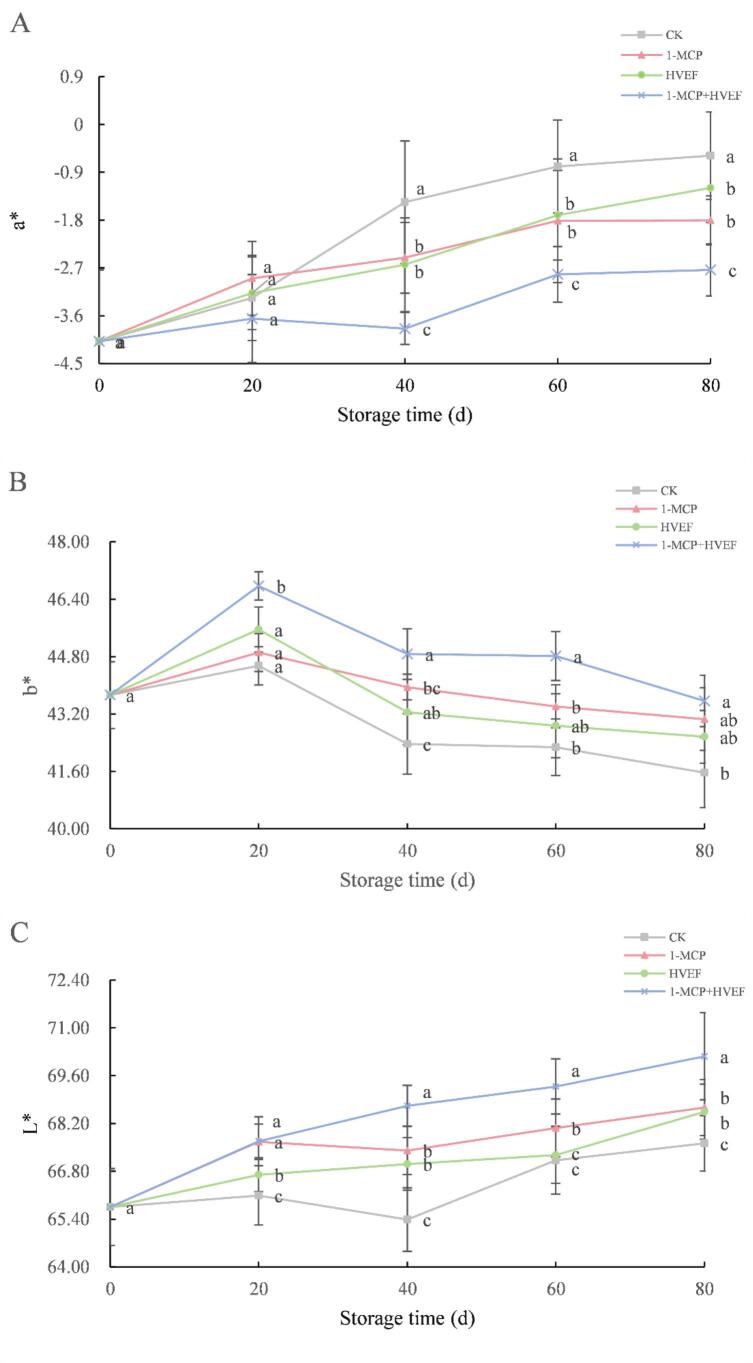
Effects of 1-MCP, HVEF, and 1-MCP + HVEF on fruit a* (A), b* (B), L* (C).

Color attributes serve as critical phenotypic indicators for evaluating the freshness and quality of fruits and vegetables. As one of the most intuitive factors influencing market value and consumer preference, color provides a reliable measure of postharvest condition (Wu et al., 2021). In this study, the color parameters of Korla fragrant pears were quantitatively assessed, where $a^{*}$ represents the green–red axis, $b^{*}$ indicates the blue–yellow axis, and $L^{*}$ corresponds to lightness. As shown in Fig. 4A and Fig. 4C during storage, the $a^{*}$ and $L^{*}$ values exhibited a continuous increasing trend, whereas the $b^{*}$ value initially increased slowly before declining gradually (Fig. 4B). Compared to the control group, the combined 1-MCP + HVEF treatment significantly suppressed the increase in $a^{*}$ value (p<0.05), indicating a delayed reddening process of the fruit. Meanwhile, the $L^{*}$ value progressively increased across all groups, suggesting enhanced surface brightness over time. At the end of storage, the 1-MCP + HVEF group showed significantly lower $L^{*}$ values than the control (p<0.05), demonstrating that the combined treatment effectively maintained fruit brightness. Additionally, a notable improvement in $b^{*}$ value was observed in the 1-MCP + HVEF group (p<0.05). These results are consistent with the color changes observed in treated cherry tomatoes (Zhao et al., 2023). The color evolution of Korla fragrant pears is primarily governed by the dynamics of chlorophyll, carotenoids, anthocyanins, and related pigments during maturation. Furthermore, respiratory activity has been shown to influence color changes in fruits and vegetables. Therefore, the delayed color alteration observed in 1-MCP + HVEF-treated pears may be attributed to the moderated respiration rate and altered pigment metabolism induced by the combined treatment.

### 3.8 Volatile flavor profiling by electronic nose

**Fig. 5:**
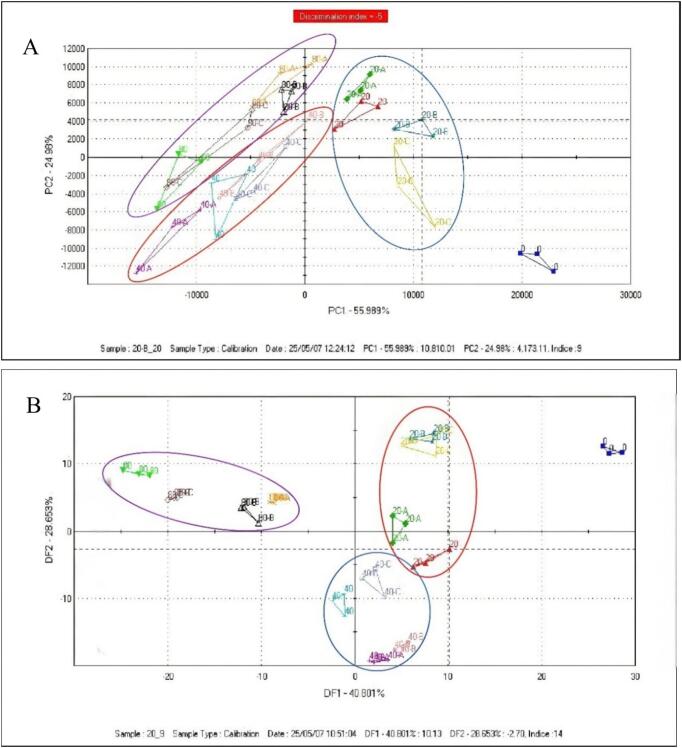
PCA and DFA of the electronic nose sensor signal response values of pear samples treated with 1-MCP, HVEF, and 1-MCP + HVEF (samples taken at 0, 20, 40, and 80 days) (A: 1-MCP treatment group; B: HVEF group; C: 1-MCP + HVEF treatment group). (A) PCA. (B) DFA.

An electronic nose (e-nose) was employed to evaluate overall flavor changes in pear samples under different postharvest treatments. This technique distinguishes samples based on their volatile compound profiles. Principal component analysis (PCA) and discriminant factor analysis (DFA) were applied to e-nose data from fruits stored for 0, 20, 40, and 80 days (Fig. 5). PCA reduces data dimensionality through linear transformation, generating a two-dimensional score plot where inter-sample distances reflect flavor dissimilarity. As shown in Fig. 5A, the first two principal components (PC1 and PC2) accounted for 55.99 % and 24.98 % of total variance, respectively, cumulatively explaining 81 % of the variation. The distinct distribution of the 13 sample groups along both axes indicated significant flavor differences among them. Day-0 samples clustered in the positive PC1/negative PC2 region, clearly separated from treated samples (20–80 days), demonstrating that all treatments substantially altered volatile profiles.

DFA was further applied to enhance inter-group separation by maximizing between-group variance while minimizing within-group variance. The first two discriminant factors explained 40.80 % and 28.65 % of the variance, respectively, with a cumulative contribution of 69 %, confirming the model's effectiveness in classifying samples by storage duration and treatment. The DFA plot (Fig. 5B) revealed clear separation between initial (0-day) and stored samples (20–80 days), consistent with PCA results. Moreover, at each storage interval, samples from the 1-MCP, HVEF, and 1-MCP + HVEF treatments formed distinct clusters with minimal overlap, indicating significant differences in their volatile compositions. Notably, the combined treatment group exhibited a flavor profile distinctly different from those of individually treated samples, highlighting a unique synergistic effect. Previous studies have shown that 1-MCP significantly modifies the volatile profile of pears, whether applied alone or in combination (Cai et al., 2019), while HVEF alone also enhances fruit aroma (Xie et al., 2025). In conclusion, the combined 1-MCP and HVEF treatment proved most effective in preserving volatile compounds and maintaining aroma quality in postharvest pears.

### 3.9 Overview of metabolome profiling

#### 3.9.1 Quantitative metabolomics analysis based on gas chromatography-mass spectrometry

**Fig. 6:**
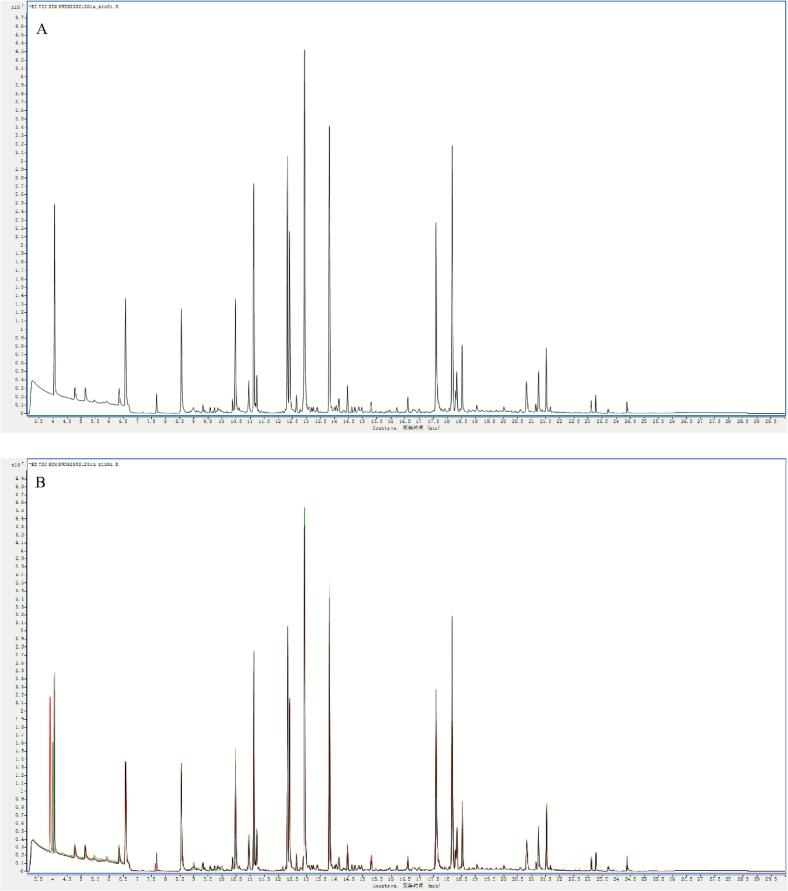

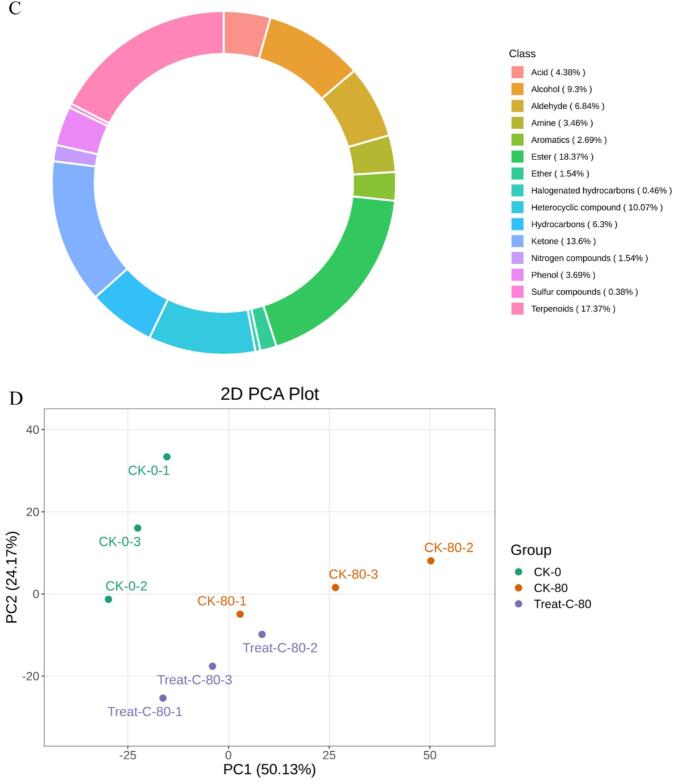

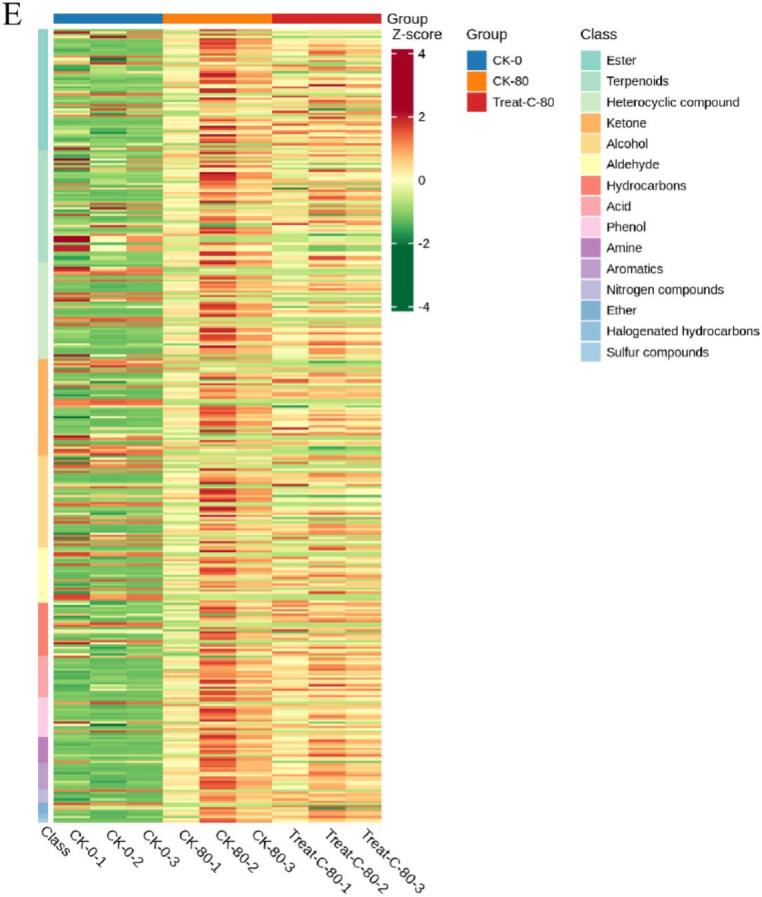

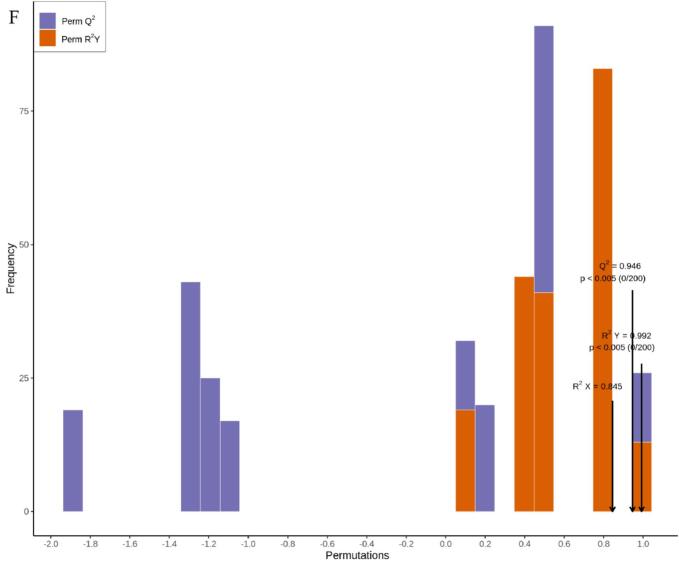

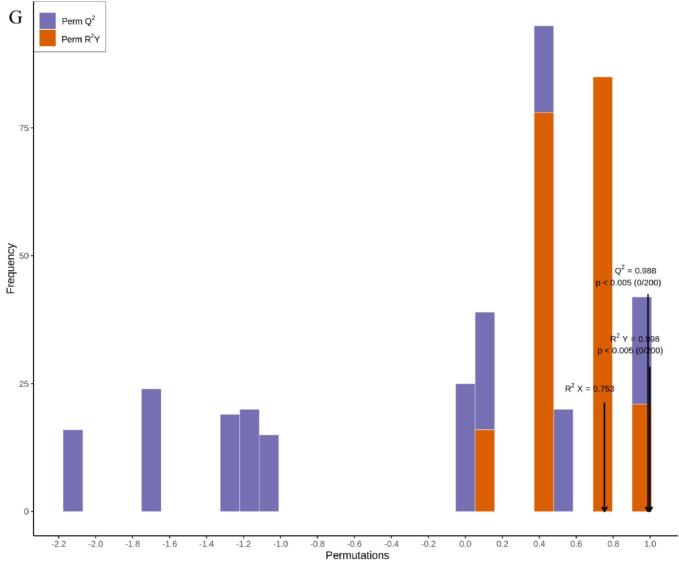

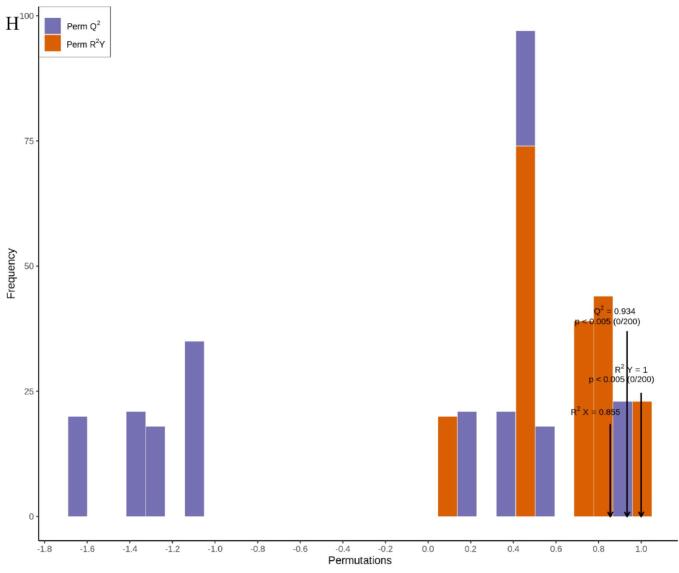
Preliminary analysis of metabolomics data. TIC of quality control samples in negative ion mode (A) and positive ion mode (B). (C) The ring graph of metabolites classes. (D) PCA plots of metabolites in CK0, CK80 and 1-MCP + HVEF 80 groups. (E) HCA of all identified metabolites. (G-H) OPLS-DA permutation analysis model verification charts of CK0 vs. CK80, CK0 vs. 1-MCP + HVEF 80 groups, CK80 vs. 1-MCP + HVEF 80 groups.

Metabolomics has gained extensive application and demonstrated its value in horticultural science through its capacity to decipher complex metabolite accumulation patterns (Wan et al., 2024). Chang et al. (2025) employed comprehensive metabolomics to identify 223 flavonoids and 202 terpenoids in jujube fruits subjected to combined treatment with acidic electrolyzed water and high-voltage electrostatic fields. This study employed HS-SPME-GC–MS for the comprehensive detection and analysis of volatile metabolites in pear samples stored for 0 and 80 days. Overlay analysis of total ion current (TIC chromatograms from mass spectrometry detection of quality control (QC) samples (Fig. 6A and B) demonstrated consistent measurements and excellent instrument reproducibility for identical QC samples. Based on the GC–MS detection platform and an in-house constructed database, 1301 metabolites across 15 classes were identified. These included esters (239), 226 terpenoids (226), ketones (177), heterocyclic compounds (131), alcohols (121), aldehydes (89), hydrocarbons (82), acids (57), phenols (48), amines (45), aromatic compounds (35), ethers (20), nitrogen-containing compounds (20), halogenated hydrocarbons (6), and sulphury-containing compounds (5) (Fig. 6C). The predominant metabolites identified in Korla fragrant pears were esters (18.37 %), followed by terpenoids (17.37 %) and ketones (13.6 %) (Fig. 6C and Table S1). Among these metabolites, 1276, 1293, and 1295 volatile organic compounds were detected in CK0, CK80, and 1-MCP + HVEF 80 samples, respectively. Two unique volatile compounds—the amine *N*-methyl-N-2-propenyl-1-butanamine (odorless), and the ether compound (Z)-1-methoxy-3-hexene (sweet, green, fruity, pear, green, apple, fresh fig), were present in the 1-MCP + HVEF 80d combined treatment group samples. These differences may be attributable to the 1-MCP + HVEF 80d combined treatment.

#### 3.9.2 Multivariate statistical analysis

To investigate differences in metabolite profiles among the CK 0, CK 80, and 1-MCP + HVEF 80 groups, unsupervised principal component analysis (PCA) and hierarchical clustering analysis (HCA), as well as supervised orthogonal partial least squares-discriminant analysis (OPLS-DA), were performed. PCA clearly revealed overall metabolic differences among the groups, as well as the degree of variation within each group. Using the identified 1301 metabolites, PCA was applied to characterize the metabolic profiles of the three sample groups. In the resulting score plot, quality control (QC) samples clustered closely with partial overlap, indicating similar metabolite profiles and supporting the reliability and reproducibility of the data. The first two principal components (PC1 and PC2) collectively explained 54.17 % of the total variance (Fig. 6D). Furthermore, the 1-MCP + HVEF-treated samples were positioned closer to the CK 0 group than to the CK 80 group, and clear segregation was observed among all three groups. A clustered heatmap was constructed based on the metabolomic data to visualize metabolite accumulation patterns across the groups (Fig. 6E). The results indicated distinct differences between the CK 80 and 1-MCP + HVEF 80 groups, a conclusion further supported by HCA. Although both the CK 80 and 1-MCP + HVEF 80 groups were stored for 80 days, the combined 1-MCP + HVEF treatment led to significant differences in the abundance of differential metabolites compared to CK 80. Notably, the metabolite profile of CK 0 differed considerably from the other two groups. These findings confirm that the metabolomic data obtained in this study are highly reproducible and suitable for subsequent in-depth analysis.

#### 3.9.3 Overview of differentially accumulated metabolites (DAMs)

OPLS-DA, a supervised modeling approach, was employed to enhance the discrimination among the CK 0, CK 80, and 1-MCP + HVEF 80 groups (Fig. 6F–H). The model parameters for the pairwise comparisons—CK 0 vs. CK 80, CK 0 vs. 1-MCP + HVEF 80, and CK 80 vs. 1-MCP + HVEF 80—yielded R2X values of 0.845, 0.753, and 0.855, respectively. Furthermore, all comparisons exhibited R2Y values above 0.99, along with high predictive ability (Q2) values of 0.946, 0.988, and 0.934. These metrics (R2Y > 0.99, Q2 > 0.9) collectively confirm the model's robustness and predictive reliability for accurately assessing inter-group differences.

**Fig. 7:**
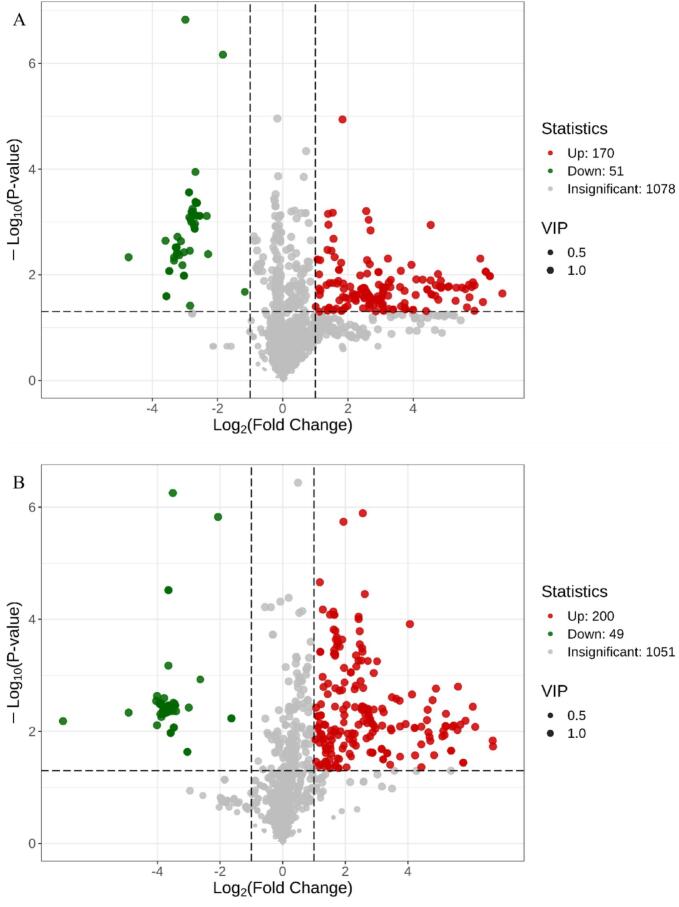

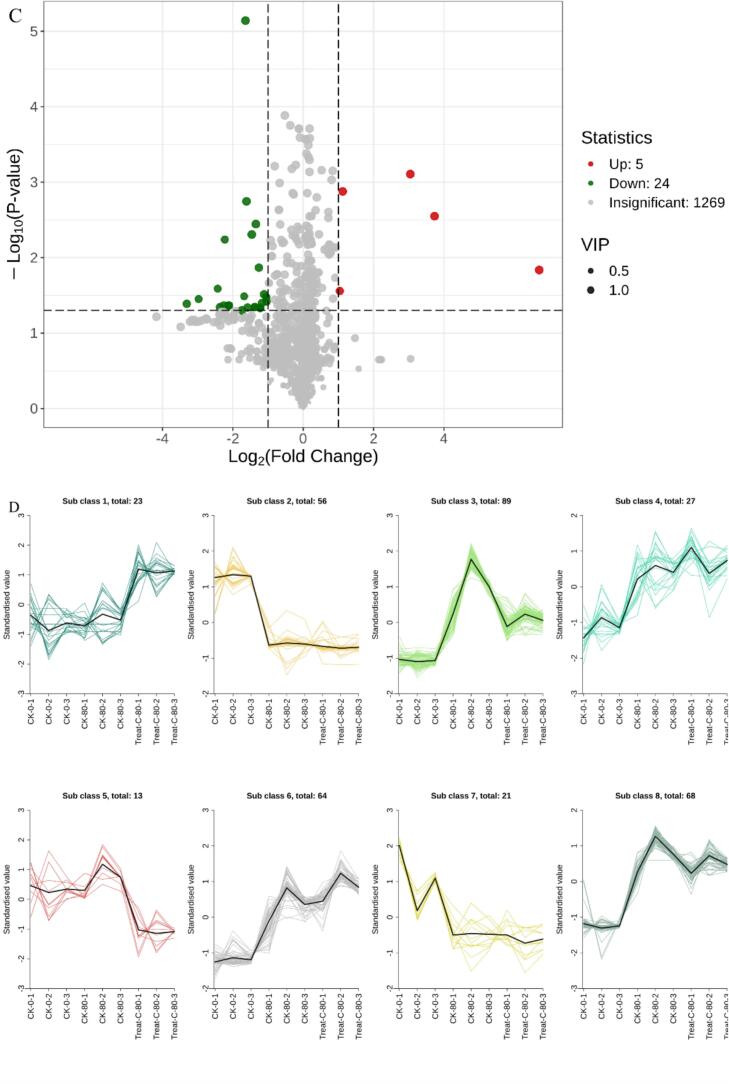

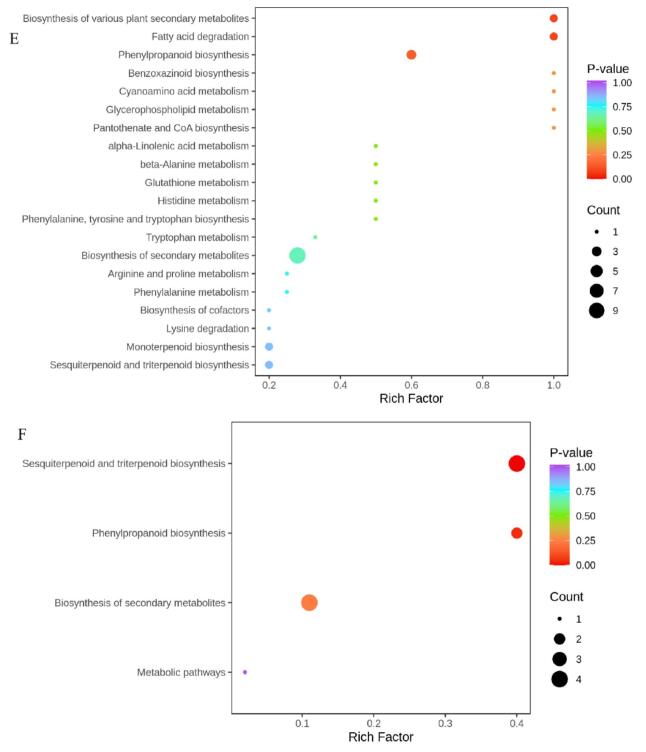

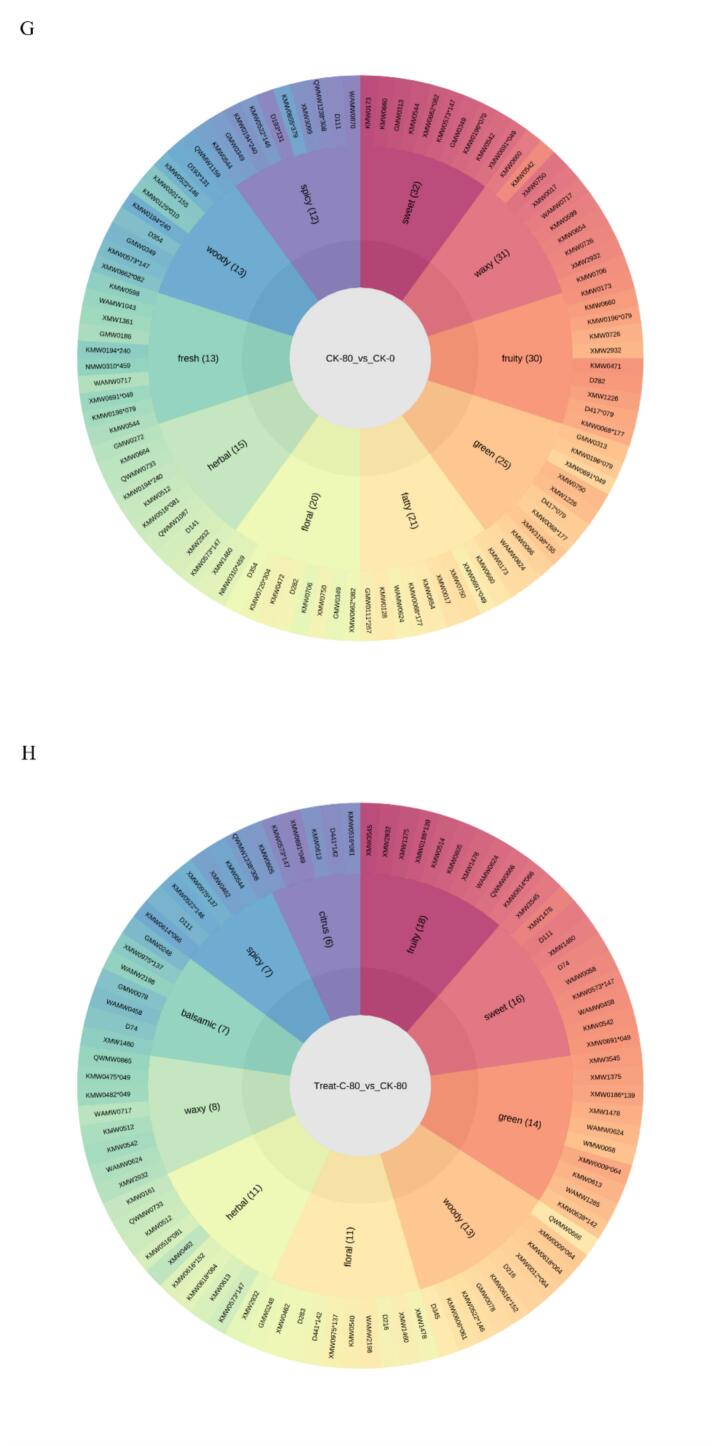
Analysis of DAMs. Volcano plots of DAMs in CK0 vs. CK80 (A), CK0 vs. 1-MCP + HVEF 80 groups (B), CK80 vs. 1-MCP + HVEF 80 groups (C). K-means Cluster Analysis of differentially accumulated metabolites in Three Different Treatment Groups of korla fragrant pear samples (D). KEGG pathways of DAMs in CK0 vs. CK80 (E) and CK80 vs. 1-MCP + HVEF 80 (F). Flavor wheel of differentially accumulated metabolites. (G), Flavor wheel of DAMs common to CK0 and CK80; and (H), Flavor wheel of DAMs common to CK80 and 1-MCP + HVEF 80.

DAMs were identified based on a fold change threshold (FC ≥ 2 or ≤ 0.5) and variable importance in projection (VIP > 1). The resulting DAMs are visualized in volcano plots and summarized in Tables S2–S4. To systematically evaluate the effects of storage duration and combined 1-MCP + HVEF treatment on Korla fragrant pears, DAMs across the three comparison groups were analyzed. As illustrated in Fig. 7A, the comparison between CK 0 and CK 80—representing the effect of storage-induced aging—revealed 221 DAMs (170 up-regulated, 51 down-regulated). In the CK 0 vs. 1-MCP + HVEF 80 comparison, which reflects the impact of combined 1-MCP + HVEF treatment, 249 DAMs were identified (200 up-regulated, 49 down-regulated) (Fig. 7B). This higher number of DAMs suggests that the combined treatment promotes the accumulation of volatile compounds during storage. Between the CK 80 and 1-MCP + HVEF 80 groups, 29 DAMs were detected (5 up-regulated, 24 down-regulated) (Fig. 7C), which are likely associated with the combination of 1-MCP and HVEF treatment. The substantially lower number of DAMs in this comparison implies that storage duration induces more extensive metabolic alterations than the combined treatment. A total of 361 DAMs were identified across all comparisons and categorized into eight classes (Fig. 7D and Table S5). Notably, 114 metabolites showed higher accumulation in the 1-MCP + HVEF-treated samples compared to both CK 0 and CK 80 (profiles 1, 4, and 6). Among these, over 31 % were terpenoids and esters (Supplementary Table S6). The metabolomic data indicate that changes in the abundance of terpenoids and esters are closely associated with flavor differences between treated and untreated samples, underscoring the key contribution of these metabolic pathways to aroma variation.

#### 3.9.4 KEGG pathway enrichment analysis of DAMs in Korla fragrant pears

KEGG database was employed to annotate and enrich the differential accumulated metabolites (DAMs) identified between the 1-MCP + HVEF combined treatment group and the control groups (CK0 and CK80). Bubble plots were used to visualize the KEGG pathway analysis of significant DAMs across the three sample groups. In the CK0 vs. CK80 comparison, DAMs were annotated to 23 metabolic pathways, with the most relevant being “Biosynthesis of various plant secondary metabolites”, “Fatty acid degradation”, and “Phenylpropanoid biosynthesis” (Fig. 7E). Between CK80 and 1-MCP + HVEF 80, four pathways were enriched, among which “Sesquiterpenoid and triterpenoid biosynthesis” and “Phenylpropanoid biosynthesis” were significantly enriched (Fig. 7F). These pathways represent precursors for terpenoid and ester biosynthesis, respectively, consistent with the observed enhancement of these volatile compounds under combined 1-MCP + HVEF treatment. Comprehensive metabolomic analysis further indicated that sesquiterpenoid and triterpenoid biosynthesis and phenylpropanoid biosynthesis are involved in the formation of key metabolites in 1-MCP-treated passion fruit (Li et al., 2020). As one of the most crucial secondary metabolic pathways in plant defense systems, phenylpropanoid metabolism plays an important role in controlling postharvest fungal diseases in fruits and vegetables. Its metabolic products have been widely documented to reduce postharvest decay in fruits (Cheng et al., 2022), while enhancing antioxidant capacity and extending shelf life.

#### 3.9.5 Sensory flavor characterization of DAMs

Aroma is a key factor influencing the sensory quality of Korla fragrant pears, closely associated with taste and nutritional attributes. Volatile composition objectively reflects the flavor profiles of different samples and serves as an important indicator for evaluating flavor quality. Sensory analysis of differentially accumulated metabolites (DAMs) was conducted to characterize the flavor attributes of pear samples. To elucidate the flavor properties of DAMs, an integrated analysis was performed using established sensory databases (https://www.odour.org.uk; https://www.flavornet.org; https://www.femaflavor.org/flavor-library). Compared to CK0, prolonged storage in CK80 resulted in a flavor profile dominated by sweet, waxy, fruity, green, fatty, floral, herbal, fresh, woody, and spicy notes (Fig. 7G). In contrast, the 1-MCP + HVEF 80 group exhibited enhanced fruity, sweet, green, woody, floral, herbal, waxy, balsamic, spicy, and citrus flavors relative to CK80 (Fig. 7H). These results indicate that fruity, sweet, green, woody, floral, herbal, waxy, and spicy attributes constitute the core flavor profile of Korla fragrant pears. The combined treatment also reduced fatty and fresh notes associated with natural senescence, while enhancing balsamic and citrus characteristics.

Secondary metabolites shape fruit aroma and significantly contribute to sensory quality. The present study identified esters and terpenoids as the most abundant volatile compounds in Korla fragrant pears, consistent with reports in ‘Nanguo’ pear (*Pyrus ussuriensis* Maxim.) (Li et al., 2023). Esters and terpenoids impart fruity and floral notes, and their decline during storage—attributed to postharvest senescence and aroma loss—aligns with previous findings (Li et al., 2016). Earlier studies also reported that 1-MCP and HVEF treatments significantly suppressed the biosynthesis of esters and terpenoids in pear (Jiang et al., 2018), and beef (Xu et al., 2020). Consistent with these reports, the combined 1-MCP + HVEF treatment reduced ester and terpenoid levels during the early storage period (Table S7). However, an opposite trend was observed at 80 d of storage: the combined treatment increased ester content from 21.482 μg/g (CK80) to 22.171 μg/g, and terpenoid content to 30.224 μg/g, restoring both to levels comparable to the initial storage stage (CK0: esters 22.834 μg/g, terpenoids 30.576 μg/g). This recovery effect contrasts with earlier studies and suggests that the combined treatment effectively preserves flavor compounds in later storage stages. During the early storage phase (0–40 d), 1-MCP combined with HVEF suppressed the emission of ester and terpenoid volatiles, consistent with the aroma-delaying effects reported for each treatment applied individually. By 80 d, however, the combined treatment led to a rebound in ester and terpenoid contents, reaching 22.171 μg/g and 30.224 μg/g, respectively—close to the initial levels and significantly higher than CK80. This phenomenon diverges from studies where individual 1-MCP or HVEF treatments continuously suppressed these volatiles over shorter storage periods. We propose that the combined treatment helps maintain overall fruit quality during extended storage. As aroma is a vital component of fruit quality and tends to diminish with metabolic decline during senescence, the synergy between 1-MCP and HVEF may reactivate biosynthetic pathways related to esters and terpenoids—such as by enhancing acetyltransferase and terpenoid synthase activities—in late storage. This could counteract the aroma loss typically induced by prolonged storage. Moreover, since most studies on individual 1-MCP or HVEF treatments focus on short- to medium-term storage, the recovery of aroma compounds at 80 d or beyond has not been previously documented. Thus, the “recovery effect” observed here may represent a novel preservation mechanism, warranting further investigation into the underlying metabolic pathways and gene regulation.

Li et al. (2023) reported that differential volatile organic compounds in “Panguxiang” pear were enriched in terpenoid biosynthesis pathways, a finding consistent with our results: 1-MCP + HVEF-treated Korla fragrant pears showed significant enrichment in the sesquiterpenoid and triterpenoid biosynthesis pathway after 80 days of storage. Thus, we postulate that the distinct balsamic and citrus flavor characteristics of Korla fragrant pears arise from the increased accumulation of terpenoids. In addition to contributing to desirable aromas, terpenoids accumulated in plants play direct or indirect roles in defense against insects and microorganisms (Ding et al., 2017). 1-MCP suppresses ethylene signaling, thereby reducing oxidative stress-derived aldehydes and acids (Jiang et al., 2024), while HVEF may physically stimulate the mevalonic acid (MVA) pathway and enhance terpene synthase activity, promoting the synthesis of terpenoid precursors. Subsequent enzymatic modifications convert these precursors into volatile aroma compounds. Together, these actions synergistically support ester synthesis pathways and help maintain the overall aromatic quality of the fruit during extended storage. We propose that the combined 1-MCP and HVEF treatment modulates terpenoid-related aroma biosynthesis through complementary mechanisms.

Although this study offers valuable insights into volatile metabolite alterations in Korla fragrant pear under combined 1-MCP and HVEF treatment, certain limitations warrant consideration. The analysis primarily targeted volatile metabolites, leaving non-volatile metabolites—including phenolics and sugars—insufficiently characterized. Moreover, the treatment was evaluated exclusively on Korla fragrant pears, and its efficacy across other pear varieties or fruit species warrants further validation. At the molecular level, the expression patterns of key enzymatic genes involved in volatile biosynthesis remain unexplored. To address these gaps, future research should incorporate non-targeted metabolomics coupled with transcriptomics to delineate the comprehensive regulatory network modulated by the 1-MCP + HVEF treatment. Extending the evaluation of this combined approach to other fruit species—such as apples and peaches—and optimizing treatment parameters for different cultivars would strengthen its general applicability. Furthermore, assessing the long-term effects of the treatment on fruit quality under shelf-life conditions following room-temperature storage would enhance its practical relevance and commercial viability.

## 4 Conclusion

In this study, an integrated approach combining physicochemical analysis and volatile metabolomics was employed to elucidate the mechanism by which combined 1-MCP and HVEF treatment improves the postharvest quality of Korla fragrant pears during storage. The results demonstrate that the combination of 1-MCP and HVEF treatment significantly suppressed respiration rate and ethylene release, maintained fruit firmness, reduced weight loss, and delayed metabolic activity, thereby effectively delaying fruit ripening and senescence. Volatile metabolomic analysis of CK0, CK80, and 1-MCP + HVEF 80 groups—which exhibited significant differences in physicochemical indices—led to the identification of 1301 metabolites via GC–MS. Esters and terpenoids constituted a substantial proportion of the detected metabolites and are likely key contributors to pear aroma. Further investigation revealed that the 1-MCP + HVEF treatment significantly altered the phenylpropanoid biosynthesis and sesquiterpenoid and triterpenoid biosynthesis pathways, promoting the accumulation of associated flavor compounds. Sensory characterization confirmed that the treatment enhanced balsamic and citrus notes while reducing fatty and fresh attributes, thereby helping to preserve the characteristic flavor profile of Korla fragrant pears. In summary, the combined 1-MCP + HVEF treatment serves as an effective strategy for maintaining postharvest quality and aroma characteristics in Korla fragrant pears, providing a theoretical basis for understanding metabolic regulation during fruit storage. However, this study primarily focused on volatile metabolites and did not fully capture dynamic metabolic changes throughout the entire storage period. Future research will integrate non-targeted metabolomics with transcriptomics to further unravel the global regulatory network and mechanisms through which 1-MCP + HVEF treatment influences flavor and quality formation in Korla fragrant pears.

## CRediT authorship contribution statement

**Yujiao Zhang:** Writing – review & editing, Writing – original draft, Visualization, Validation, Methodology, Investigation, Formal analysis, Conceptualization. **Chengxin Fei:** Writing – review & editing, Validation, Investigation, Data curation. **Ruojie Zhao:** Writing – review & editing, Validation, Investigation, Data curation. **Oujun Dai:** Writing – review & editing, Validation, Investigation, Data curation. **Zhixiong Deng:** Writing – review & editing, Validation, Investigation, Data curation. **Bei Fan:** Supervision, Project administration, Funding acquisition. **Duoyong Zhao:** Project administration, Funding acquisition. **Fengzhong Wang:** Supervision, Project administration, Funding acquisition. **Yatao Huang:** Writing – review & editing, Supervision, Software, Project administration, Funding acquisition.

## Funding

This work was supported by the Project of Fund for Stable Support to Agricultural Sci-Tech Renovation (xinkywdzc-2024002-05); Tingzhou Outstanding scientific and technological talents project; Xinjiang Uygur Autonomous Region” Tianchi Talent” Training Plan Project, “Tingzhou Talents” Innovation Team [2023CT02]; Agricultural Science and Technology Innovation Program of Chinese Academy of Agricultural Sciences.

## Declaration of competing interest

The authors declare that they have no known competing financial interests or personal relationships that could have appeared to influence the work reported in this paper.

## Data Availability

Data will be made available on request.

## Appendix A Supplementary data

Supplementary material Image 1
